# Role of Vascular Endothelial Growth Factor and Human Umbilical Vein Endothelial Cells in Designing An *In Vitro* Vascular-Muscle Cellular Model Using Adipose-Derived Stem Cells

**DOI:** 10.22074/cellj.2020.7034

**Published:** 2020-09-08

**Authors:** Abbas Heidari-Moghadam, Vahid Bayati, Mahmoud Orazizadeh, Mohammad Rashno

**Affiliations:** 1. Cellular and Molecular Research Center, Ahvaz Jundishapur University of Medical Sciences, Ahvaz, Iran; 2. Department of Anatomical Sciences, Faculty of Medicine, Ahvaz Jundishapur University of Medical Sciences, Ahvaz, Iran; 3. Department of Immunology, Faculty of Medicine, Ahvaz Jundishapur University of Medical Sciences, Ahvaz, Iran

**Keywords:** Human Umbilical Vein Endothelial Cells, Mesenchymal Stem Cells, Myogenic Differentiation, Vascular Endothelial Growth Factor

## Abstract

**Objective:**

Researchers have been interested in the creation of a favorable cellular model for use in vascular-muscle tissue engineering. The main objective of this study is to determine the myogenic effects of vascular endothelial growth factor (VEGF) and human umbilical vein endothelial cells (HUVECs) on adipose-derived stem cells (ADSCs) to achieve an *in vitro* vascular-muscle cellular model.

**Materials and Methods:**

The present experimental research was conducted on two primary groups, namely ADSCs monoculture and ADSCs/HUVECs co-culture that were divided into control, horse serum (HS), and HS/VEGF differentiation subgroups. HUVECs were co-cultured by ADSC in a ratio of 1:1. The myogenic differentiation was evaluated using the reverse transcription-polymerase chain reaction (RT-PCR) and immunofluorescence in different experimental groups. The interaction between ADSCs and HUVECs, as well as the role of ADSCs conditional medium, was investigated for endothelial tube formation assay.

**Results:**

Immunofluorescence staining indicated that Tropomyosin was positive in ADSCs and ADSCs and HUVECs co-culture groups on HS and HS/VEGF culture medium. Furthermore, the MyHC2 gene expression significantly increased in HS and HS/VEGF groups in comparison with the control group (P<0.001). More importantly, there was a significant difference in the mRNA expression of this gene between ADSCs and ADSCs and HUVECs co-culture groups on HS/VEGF culture medium (P<0.05). Current data revealed that the co-culture of ADSCs and HUVECs could develop endothelial network formation in the VEGF-loaded group. Also, the ADSCs-conditioned medium improved the viability and formation of the endothelial tube in the HS and VEGF groups, respectively.

**Conclusion:**

It was concluded that ADSCs/HUVECs co-culture and dual effects of VEGF can lead to the formation of differentiated myoblasts in proximity to endothelial network formations. These *in vitro* cellular models could be potentially used in vascular-muscle tissue engineering implanted into organ defects where muscle tissue and vascular regeneration were required.

## Introduction

Skeletal muscle tissue plays a major role in body movement, keeping posture, and supporting the skeletal system that accounts for about half of human body weight (1). Due to its specific resident progenitor cells, called satellite cells, this tissue has an innate capacity to self-repair after injury (2). It should be noted that these cells have limited capacity; hence, in the case of intensive damage to muscle tissue (which destroys the basal membrane of cells), it is stem cells cannot repair the damaged area and remains as a permanent disability (3). The specific tissue damage called "Volumetric Muscle Loss" (VML) was considered to define the research pathway. VML is irreversible damage (both volumetric and contractile) of muscle tissue and can occur because of tumors, surgery, accidents, etc. (4, 5). It is found that there are insufficient treatment options of VML, and there is no proper, effective, and promising treatment for it (5). In recent years, new methods, such as regenerative medicine with its high potential for replacing and repairing tissues and organs have been promising for researchers to solve these problems (6). Designing in-vitro favorable cellular models for use in tissue engineering can improve the therapeutic choices for numerous serious health challenges, such as VML (7). Since two decades ago, the use of stem cells in research has attracted much attention. A specific stem cell called adipose-derived stem cell (ADSC) has been gradually introduced with increasing research (8, 9). These cells are ideal options in regenerative medicine because of features such as the ease of isolation and cultivation and their control, high ability of growth and differentiation, nonaggression, and finally, the absence of ethical issues about them (9). Creating an ideal cellular composition for differentiation and forming of myofibrils along with the development of vascular and neural structures have always been complicated and challenging. The co-culture of endothelial cells with other types of cells is a new approach to construct a vascular cellular composition for use in tissue engineering (10). For instance, the co-culture of endothelial cells with fibroblasts (11), primary osteoblasts, or smooth muscle cells have been able to significantly increase vascular sprouts and structures *in vitro* (11, 12). The vascular endothelial growth factor (VEGF) is a key factor and regulator of embryonic angiogenesis. This factor has inductive effects on endothelial cells (in the creation of vascular networks) (13), neurons, hepatocytes, and myoblast in stimulating cell migration, protecting them against apoptosis, and inducing myoblasts to form muscle fibers (14, 15). Therefore, the present study was designed to determine the myogenic effects of VEGF and human umbilical vein endothelial cells (HUVECs) on ADSCs to achieve an ideal cellular model and create an *in vitro* differentiated vascularmuscle structure.

## Materials and Methods

### Adipose-derived stem cells isolation and culture

In this experimental study, isolation of ADSCs was performed based on previous protocols (16). So, after excision, the gonadal fat tissues of Wistar rats were washed three times with phosphate-buffered saline (PBS, Invitrogen, USA), containing 1% penicillin/ streptomycin (Gibco, USA) to remove blood vessels and debris, and then cut into small pieces to facilitate enzymatic digestion. Samples were incubated with 0.1% collagenase type I at 37°C for 30-50 minutes and then neutralized enzymatic activity by adding cell culture media Dulbecco's Modified Eagle Medium (DMEM, Invitrogen, USA), 10% fetal bovine serum (FBS, Sigma, USA) to the solution. For the separation of mature adipocytes from the remaining stromal-vascular fraction (SVF), the cellular mixture was centrifuged at 2000 rpm for 5 minutes. The supernatant was removed, and the cell plate was resuspended in 3 ml growth media containing (DMEM) supplemented with 10% FBS, 1% L-glutamine, and 1% penicillin/streptomycin. SVF cells were plated at 2.5×10^4^ cells/cm^2 ^per 25 cm^2^ cell culture flasks and incubated at 37˚C in 5% CO_2_. The non-adherent cells were removed after the substitution of the cell culture medium following 48 hours. At passage 4, the surface antigens of cells, including CD44, CD73, and CD90 as positive markers and CD45 as a negative marker, were evaluated for ADSCs characterization by flow cytometry assay. To promote myogenic differentiation, determined ADSCs were cultured in DMEM containing 10% FBS and 3_M 5-Azacytidine (Sigma, NY, USA) for 24 hours and then in DMEM supplemented with 5% horse serum (HS, Gibco, NY, USA) for 7 days. Supplemented media was replaced every 48 hours.

### Ethical consideration

This study was approved by the Ethics Committee of Ahvaz Jundishapur University of Medical Sciences (IR. AJUMS.REC.1396.282). All protocols, such as animal care, anesthesia, and euthanasia procedures, were performed in accordance with the guidelines of the Ethics Committee of Ahvaz Jundishapur University of Medical Sciences.

### Co-culture models

HUVECs were purchased from the National Center for Genetic and Biological Resources (Tehran, Iran) and cultured in DMEM containing Ham’s F12 (1:1), 20% FBS, 10 mM L-Glutamine, 2 mM Sodium Pyruvate, 2 mM nonessential amino acids, 0.5 μg/ml hydrocortisone and 50 μg/ml ascorbic acid with 5% CO_2_ and 37°C after being labeled with Cell Tracker TM CM-DiI (C7000, Sigma, NY, USA) according to its instructions.

In the present study, we designed two primary experimental groups in order to investigate the roles of endothelial cells and VEGF165 (Sigma, NY, USA) on the differentiation of ADSCs as follows:

I. ADSCs

II. ADSCs and HUVECs co-culture

Each group was then divided into 3 subgroups including:

1. Control group (C) in which cells were cultured in DMEM containing Ham’s F12 (1:1), supplemented with 10% FBS, 1% L-glutamine and 1% penicillin/ streptomycin

ADSCs/DMEM

ADSCs/HUVECs/DMEM

2. Differentiation group with HS, cultured in the differentiation media (HS and *5-azacytidine*)

ADSCs/HS

ADSCs/HUVECs/HS

3. Differentiation group with HS and VEGF cultured in differentiation media (HS and *5-azacytidine*+50 ng/μl VEGF)

ADSCs/HS/VEGF

ADSCs/HUVECs/HS/VEGF

**Fig.1:**
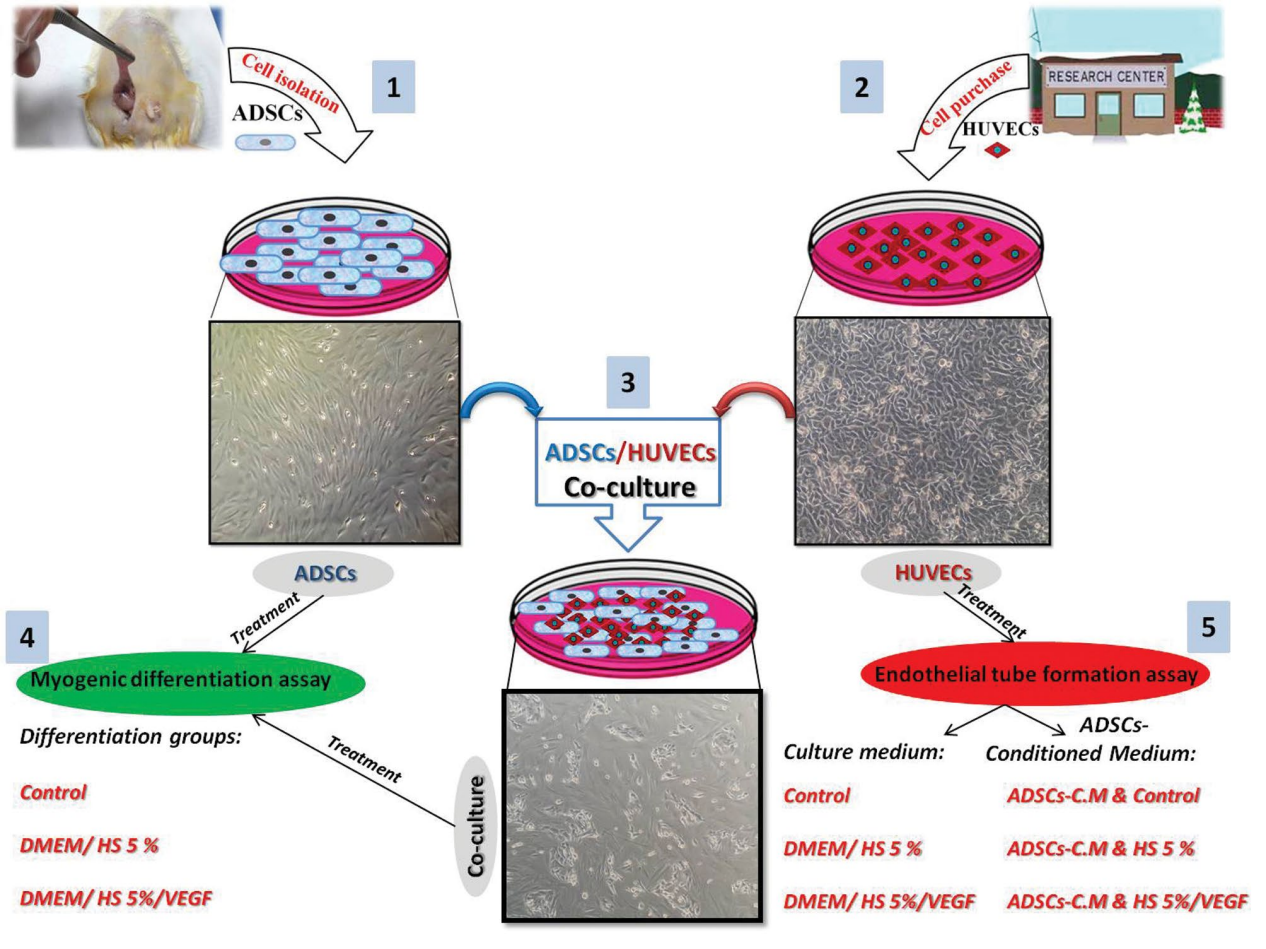
The schematic diagram shows the experimental procedures. Adipose-derived stem cells (ADSCs) and human umbilical vein endothelial cells (HUVECs) cultivation (step 1 and 2) and ADSCs/HUCEVs co-culture (step 3) was described. Step 4 and 5 represents the experimental groups. In this research 3 types of culture medium including myogenic differentiation and ADSCs-conditioned medium (ADSCs-C.M) were designed (step 4 and 5). Myogenic differentiation assay in ADSCs and co-culture groups were evaluated, separately (step 4). And finally, in step 5, HUVECs were treated by different types of culture mediums to examine the endothelial tube formation.

HUVECs were co-cultured with ADSC in a ratio of 1:1. Cells were treated with 50 ng/μl VEGF for VEGF *in vitro* experiments. Supplemented media was replaced every 48 hours. Figure 1 summarizes the main points of the experiment.

In addition to direct the interaction of ADSCs and HUVECs in the present research, it was also evaluated the role of the conditioned medium of ADSCs (CMADSCs) on the growth, proliferation and endothelial network formation of HUVECs. In this regard, HUVECs were divided into two primary groups: HUVECs and induced HUVECs (iHUVECs) groups. Each group was then divided into 3 subgroups as follows:

1. HUVECs/DMEM

2. HUVECs/HS

3. HUVECs/HS/VEGF

4. HUVECs/ADSCs-C.M/ DMEM

5. HUVECs/ADSCs-C.M/ HS

6. HUVECs/ADSCs-C.M/ HS/VEGF

In the iHUVECs group, cells were induced by the ADSC-conditioned medium (ADSC-CM) and HUVEC special culture medium at a ratio of 1:1.

### Cell labeling

Endothelial cells were marked with CM-DiI (C7000) according to the manufacturer’s instructions. Labeled- HUVECs fluorescence was confirmed by microscopy after 24 hours.

### Real-time reverse transcriptase-polymerase chain reaction analysis

We used real-time reverse transcriptase-polymerase chain reaction (RT-PCR) to confirm the expression of *MyHC2* with ABI (STEP1, USA) according to the manufacturer’s instructions. So at first, for RNA extraction, the cells were lysed using the RNeasy Plus Mini Kit (Qiagen, Gaithersburg, MD, USA) in Eppendorf tubes. Then the cells were quantified by using a NanoDrop 2000c spectrophotometer (Thermo Scientific, USA). cDNA synthesis was performed using a QuantiTect Reverse Transcription Kit (Qiagen, Gaithersburg, MD, USA). The following primer sequences for amplification were used:

*MyHC2*-

F: 5′-GGCTGGCTGGACAAGAACA-3′

R: 5′-CCACCACTACTTGCCTCTGC-3′

GAPDH

F: 5′-TGCTGGTGCTGAGTATGTCGTG-3′

R: 5′-CGGAGATGATGACCCTTTTGG-3′

### Immunofluorescence analysis

The cells were washed 3 times with PBS and then with 4% paraformaldehyde were fixed (Sigma, USA) for 20 minutes, washed with PBS and permeable with Triton X-100 (Merck, USA) for 10 minutes, washed again with PBS, subsequently; the cells were incubated with 3% bovine serum albumin (BSA, Sigma, USA) for 2 hours to block any non-specific binding.

ADSCs and ADSCs/HUVECs experimental groups were stained with a primary antibody against the anti-tropomyosin antibody (1:100, Sigma, USA) overnight at 4˚C. Then, the specimens were rinsed three times with PBS and incubated with goat anti-mouse fluorescein isothiocyanate (FITC)- conjugated secondary antibody (1:150, Sigma, USA) for 1 hour. Nuclear staining was done with 4´, 6-diamidino-2- phenylindole (DAPI, 1:400, Sigma, USA) for 15 minutes at room temperature. Ultimately, the plates were washed three times with PBS and then examined by an invert fluorescent microscope (IX 71, Olympus, Japan).

### Adipose-derived stem cell-conditioned medium preparation:

ADSCs were cultured in a complete culture medium when reached confluence, and then the medium was replaced with a serum-free or low serum-containing medium. After changing the medium, ADSCs cells were cultured under hypoxia (2% O_2_, 5% CO_2_) for 48 hours. The conditioned media of ADSCs were collected, centrifuged at 2,000 rpm and 4°C for 10 minutes, and finally filtered using a 0.22 mm syringe filter.

### Endothelial tube formation assay

HUVEC tube formation was analyzed using the Angiogenesis Analyzer of Image J software (version 1.47 from [http://imagej.nih.gov/ij/]). Therefore, the tube formation was calculated by counting numbers of connected cells (meshed network) in random fields with an inverted microscope and by dividing by the total number of cells in the same field.

### Statistical analysis

The whole data was presented as the mean ± standard deviation of three triplicated independent experiments. To meet study objectives, data were analyzed using different techniques, including one-way analysis of variance (ANOVA) followed by Tukey’s post hoc test for each paired experiment. All analyses were done using GraphPad Prism Software (version 5.1, Graphpad Software Inc., La Jolla, CA, USA). Moreover, P<0.05 was *considered* statistically significant.

**Fig.2:**
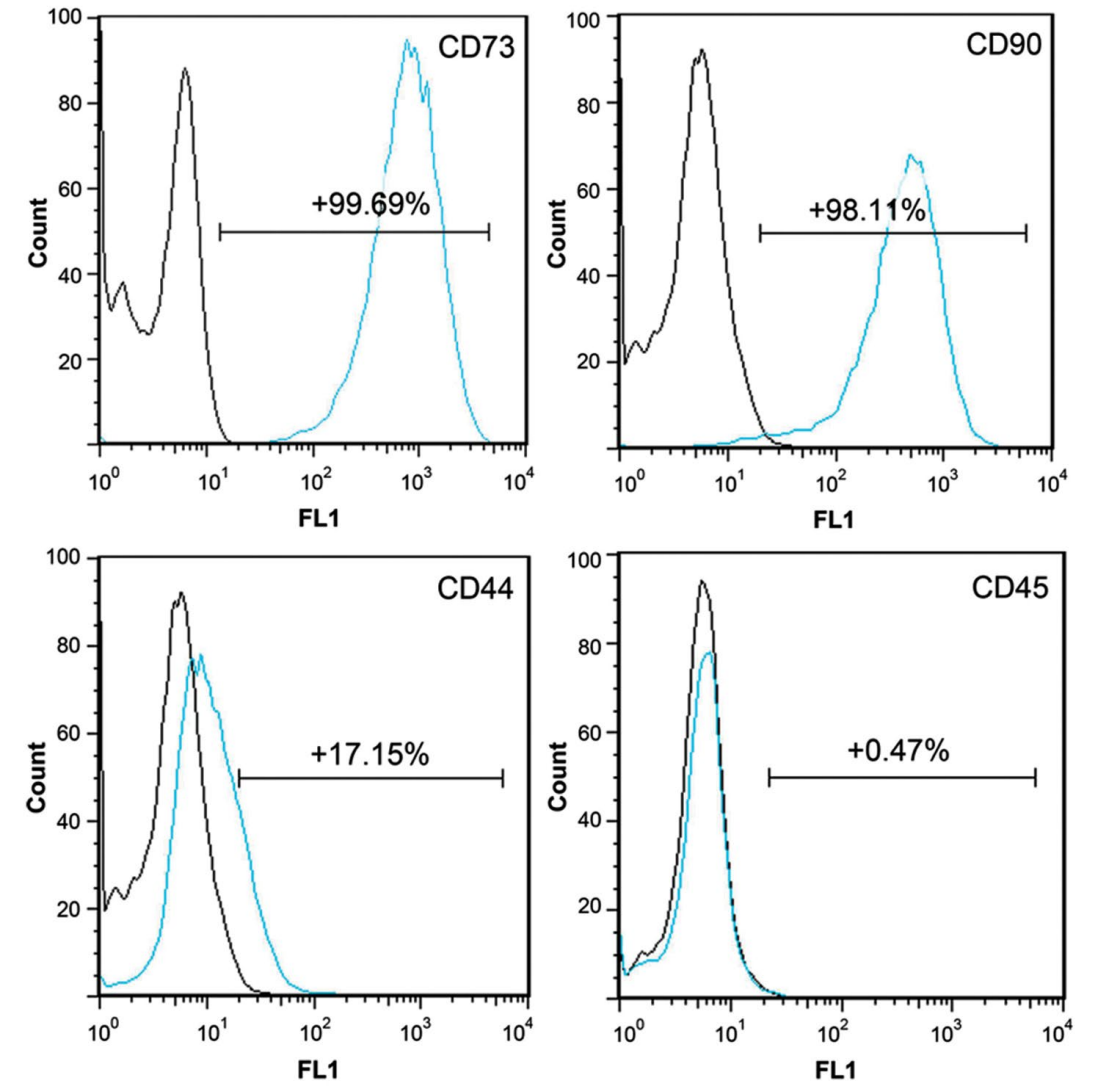
Flow cytometric analysis of cell surface marker expression in adipose-derived stem cells (ADSCs) at 4^th^ passages. Histograms represent the positive mean value of each marker.

## Results

### Characterization of adipose-derived stem cells

Flow cytometric analysis of passage 4 ADSCs revealed that CD73 (99.69%), CD90 (98.11%), and CD44 (17.15%) were expressed on the cell surface as a positive marker of mesenchymal stem cells. however, only in a few cells, CD45 (0.47%) was expressed as a negative marker (Fig .2).

### Changes in gene mRNA expression

**Fig.3:**
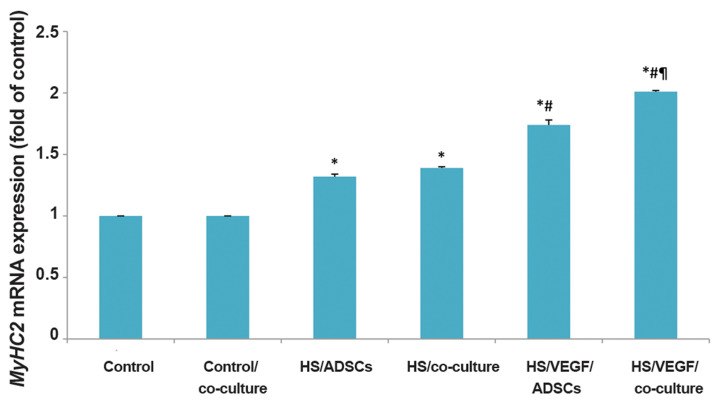
Expression of *MyHC2* mRNA in experimental groups. *MyHC2* mRNA expression increased 1.32 ± 0.02, 1.39 ± 0.01, 1.74 ± 0.04, 2.01 ± 0.01 fold in HS/ADSCs, HS/co-culture, HS/VEGF/ADSCs and HS/VEGF/co-culture groups, respectively. Data are mean ± SD of 3 separate experiments. *, #, and ¶ symbols indicate comparison to control, HS and HS/VEGF groups, respectively. ADSCs; Adipose-derived stem cells, VEGF; Vascular endothelial growth factor, HS; Horse serum, *; P<0.001, #, and ¶; P<0.05.

Quantitative real-time RT-PCR demonstrated that mRNA expression of *MyHC2* increased significantly in the HS and HS/VEGF groups compared to the control group (P<0.001). There was a significant difference in the mRNA expression of this gene between HS/VEGF and HS group (P<0.05). Most importantly, *MyHC2* expression was *significantly upregulated* on HS/VEGF in the co-culture group compared to the monoculture group (P<0.05). However, the observed gene expression difference was not significant between ADSCs and coculture groups on HS (Fig .3).

These results showed that VEGF with HUVECs upregulates the expression of *MyHC2* in differentiated ADSCs compared to other groups.

### Immunofluorescence assay

**Fig.4:**
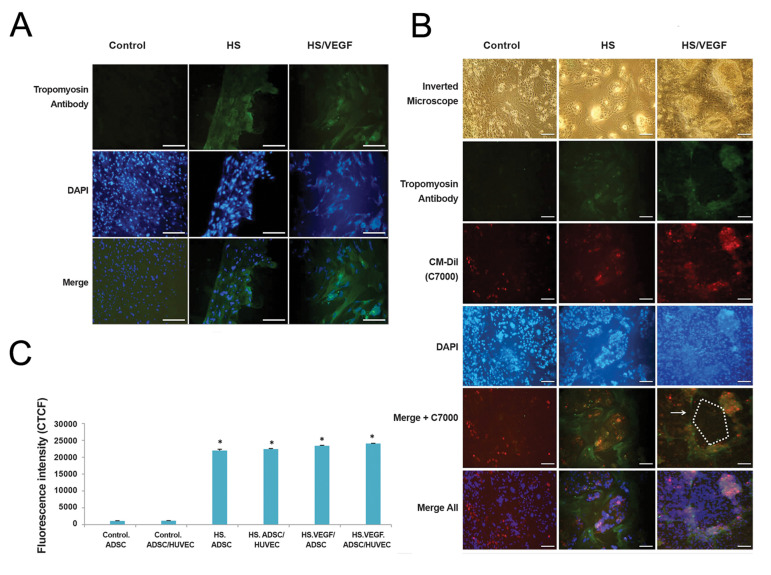
Immunofluorescence and Morphological characterization in experimental groups. (**A.** ADSCs and **B.** ADSCs/HUVECs). Immunofluorescence confirmed the expression of tropomyosin in HS and HS/VEGF groups. Negative control groups were cultured in proliferation (not differentiation) medium and set using both primary and secondary antibodies. The results showed no expression of tropomyosin in these groups. ADSCs were arranged in proximity to endothelial cells and looked like the vascular network in the HS/VEGF co-culture group. Arrows and schematic shapes represent endothelial tube formation. C. CTCF assessment of tropomyosin antibody in experimental groups. CTCF in the HS and HS/VEGF groups was significantly increased in comparison to the control group. However, the index of HS/VEGF group showed that there was no significant difference compared to the HS group. * symbol indicates comparison to control groups (scale bare: **A:** 500 μm and** B:** 200 μm). ADSCs; Adipose-derived stem cells, HUVECs; Human umbilical vein endothelial cells, HS; Horse serum, VEGF; Vascular endothelial growth factor, CTCF; Corrected total cell fluorescence, and *; P<0.001.

Immunofluorescence staining showed that the expression of tropomyosin in HS and HS/VEGF differentiation groups was positive compared to the control group (Fig .4A, B). According to the previous protocol, fluorescence intensity measurements were evaluated with image J software (17). As shown in Figure 4C, Corrected Total Cell Fluorescence (CTCF) in HS and HS/VEGF groups was significantly higher than the control group (P<0.001). However, there was no significant difference between HS and HS/VEGF groups (Fig .4C).

In the ADSC groups, the orientation of differentiated cells due to HS was arranged in dense parallel form, while the myoblast-like cells did not have any uniform arrangement on HS/VEGF, which probably due to the role of VEGF in the promotion of ADSCs towards the vascular, skeletal, muscle tissues (Fig .4A).

In the co-culture groups, it seems that the shape and orientation of ADSCs were impressed by two factors: VEGF and HUVEC cells. So that, in the control coculture group, ADSCs were distributed between the endothelial cells, While, differentiated ADSCs were situated between the endothelial cell colonies (cellular islets) in the HS co-culture group. More interestingly, these cells were arranged in proximity to endothelial cells and looked like the vascular network in the HS/VEGF coculture group (Fig .4B).

These results suggested that HS/VEGF-loaded medium has dual effects (Myogenesis, Angiogenesis) on both ADSCs monoculture and ADSCs/HUVECs co-culture.

### Endothelial tube formation assay

**Fig.5:**
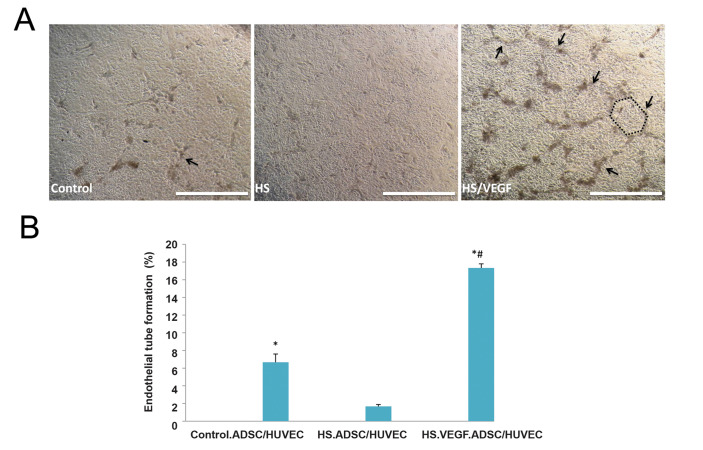
Endothelial tube formation assay (ETFA) in experimental co-culture groups. **A, B.** ETF in the control group indicated a significant increase compared to the HS group. Also, ETF was significantly increased in HS+VEGF group compared to other groups (arrows and schematic shapes represent ETF). *, # symbols indicate comparison to HS and control groups, respectively (scale bare: 1 mm). ADSCs; Adipose-derived stem cells, HUVECs; Human umbilical vein endothelial cells, HS; Horse serum, VEGF; Vascular endothelial growth factor, *, and #; P<0.001.

It is interesting to note that besides myogenic differentiation, endothelial tube formation of HUVECs was observed by inverted microscopy in the present study. The endothelial tube formation was quantified by measuring total capillary tube length using image j software. Our findings indicated that the endothelial tube formation was significantly higher than the HS group in HS/VEGF and control co-culture groups (P<0.001). Moreover, there was a significant difference between HS/VEGF and the control group (P<0.001, Fig .5A, B).

The results indicated the endothelial tube formation of HUVECs in control, and more importantly, VEGF-loaded culture media.

### Conditional medium assay

It was found that HUVECs had an optimal growth in the HS culture medium in co-culture when compared to the culture of HUVECs alone on the HS medium. Therefore, it seems that the ADSCs culture medium (conditioned medium) has an effect on endothelial cell growth.

**Fig.6:**
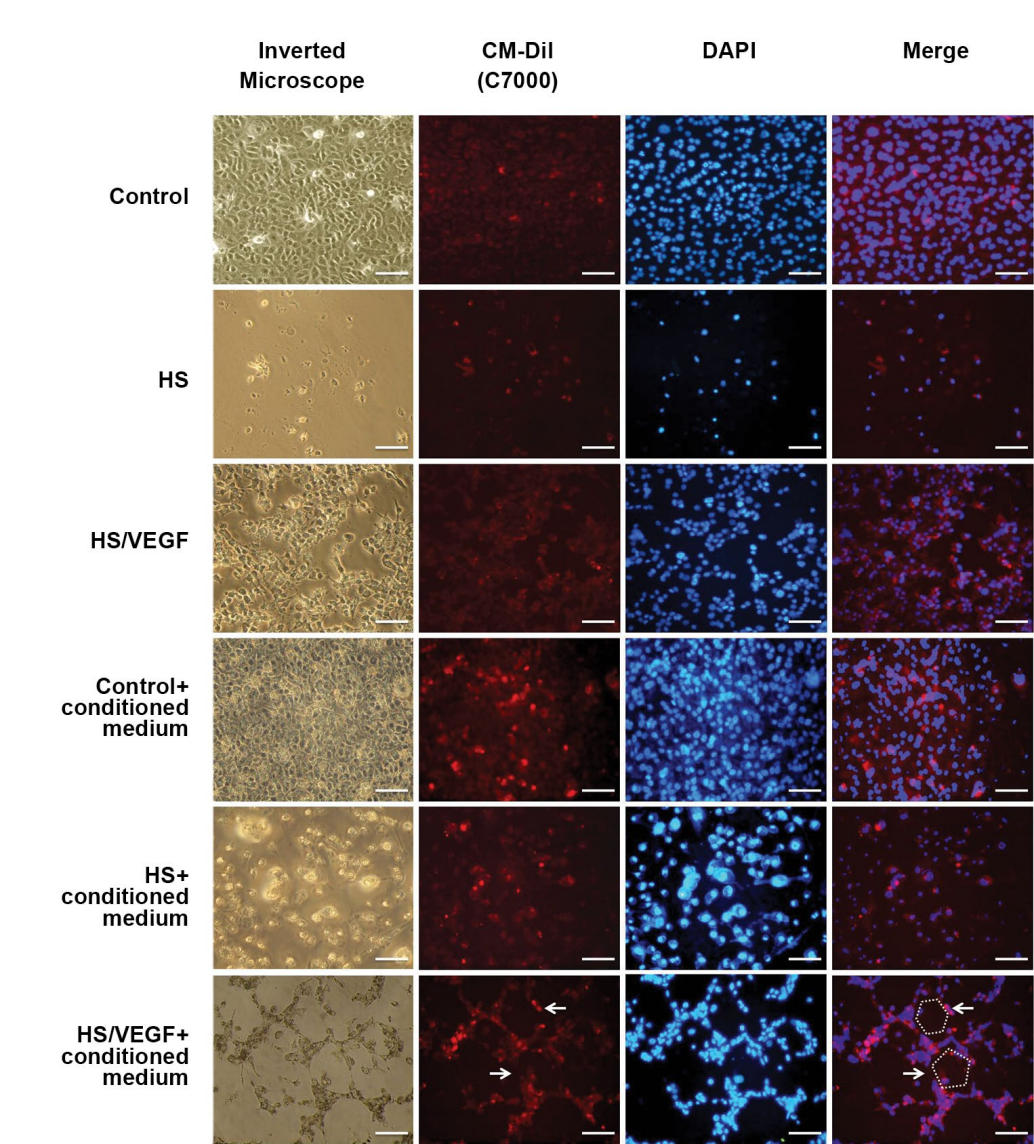
ADSCs conditioned medium effects on HUVECs orientation in experimental groups. In control groups, the cells kept their natural growth in both culture medium and ADSCs-CM. In the HS groups, ADSCs-CM prevented HUVECs from cell death and apoptosis. It can be observed from the figure that endothelial tube formation was promoted in HS/VEGF ADSCs-CM in comparison to other groups (arrows and schematic shapes represent ETF, scale bare: 300 μm). ADSCs-CM; Adipose-derived stem cells-conditioned medium, HUVECs; Human umbilical vein endothelial cells, HS; Horse serum, and VEGF; Vascular endothelial growth factor.

According to the conditioned medium of ADSCs in the control group, it was found that the cells retained their natural growth in the culture medium, but no endothelial tube formation was observed. In the HS group, the conditioned medium prevented HUVECs from cell death and apoptosis. Figures showed a remarkable improvement in the cell viability compared to the primary culture medium, while the conditioned medium considerably promoted the endothelial tube formation in the HS/VEGF group after 3 days (Fig .6). Our data indicated that HS/ VEGF ADSCs-CM could induce the endothelial tube (network) formation in HUVECs monoculture.

## Discussion

It is now known that skeletal muscle tissue engineering is a complex process that requires the formation of myofibers, construction of functional vasculature, innervation, and an improvement in the extracellular matrix (ECM) that allows for the production of a proper mechanical force (18). In the present experimental research, we investigated the effects of HUVECs and VEGF on myoblast differentiation of ADSCs to achieve acceptable and functional cellular models for use in muscle tissue engineering. In this regard, ADSCs were cultured in a 2D system alone or with HUVECs with or without VEGF in the culture medium. ADSCs/HUVECs were co-cultured in a ratio of 1:1. This ratio was based on previous studies that have been used between ADSCs/HUVECs (19) and myoblast/HUVECs (20).

Results of current research indicated that VEGF could improve the myogenic differentiation of ADSCs in monoculture or co-culture groups. VEGF is a key regulator of angiogenesis, but it is not clear whether it would restore muscle force and aid in muscle regeneration after acute musculoskeletal injuries. Recent studies suggest that VEGF may affect a variety of other cell types such as neurons, hepatocytes, osteoblasts, hematopoietic cells, and myoblasts (13). Moreover, it was demonstrated that VEGF administration in vitro stimulates myoblast migration and survival, protects myogenic cells from apoptosis, and promotes myogenic cell growth (14, 15). Chen et al. (21) and Song et al. (22) in separate studies reported that VEGF was responsible for the cardiomyocyte differentiation of ESCs and ADSCs, respectively.

Furthermore, Kim et al. (23) reported that the VEGF, when combined with ADSCs, could be used as a vascularizing tool for tissue engineering of complex muscle tissue. Based on our investigation, VEGF induced the endothelial tube formation in HS/VEGF co-culture groups besides myoblast differentiation of ADSCs. These data support the idea that appropriate myogenesis will occur along with angiogenesis, and VEGF could promote both of them. It has been suggested that the increased angiogenesis induced by VEGF might improve the muscle function in ischemic tissues (14).

The present findings were consistent with other studies that identified a relationship between angiogenesis and more effective muscle regeneration (10, 24, 25). According to previous studies, in addition to VEGF, it seems necessary to utilize endothelial cells to create ideal vasculature and skeletal muscle (26, 27).

In the present research, ADSCs were co-cultured with HUVECs to investigate myoblast differentiation in stem cells. In this regard, we assessed direct cell-cell interaction and paracrine effects on differentiation, morphology, and directions of cells. It was based on our hypothesis that direct cell-cell interactions between cells probably enhanced the myoblast differentiation.

In this study, it has been demonstrated that HUVECs myogenic effects are impressive when utilized along with VEGF and can promote the development of a favorable vascular-muscle structure. Koffler et al. (28) sought to cultivate muscle progenitor cells (MPCs), endothelial cells (ECs), and fibroblasts on an acellular biological scaffold that was used for abdominal wall defect in nude mice. It was determined that the co-culture of myoblast cells and HUVECs in a sandwich structure could improve vascular formation (29).

The co-culture of ECs with MPCs, in addition to increased angiogenesis, could improve the formation of muscle tissue (18). Previous research found that the VEGF secretion by ECs resulted in the migration of MPCs and protection against apoptosis (15). It is believed that endothelial cells can induce smooth muscle cell differentiation in bone marrow stem cells (BMSCs). It is reported that ECs can promote a contractile phenotype, reduce proliferation, and increase the synthesis of collagen (30). There is insufficient data about ADSCs/ HUVECs co-culture in the myogenic differentiation. An advantage of the current study is that it investigated the role of HUVECs in myogenic differentiation as well as the orientation of ADSCs in a co-culture model. As mentioned in previous publications, the myogenesis occurred along with the angiogenesis. Therefore, the endothelial tube formation was evaluated *in vitro* in the present study. We hypothesized that the interaction between ADSCs and HUVECs might lead to the proliferation, improvement of the cellular arrangement, and angiogenesis of endothelial cells, and this process could mutually improve the myoblast differentiation. It is believed that ECs were induced to form capillary-like structures during the reorganization stage of angiogenesis *in vitro*.

It has been reported that ADSCs/ECs co-culture can induce endothelial tube formation and significantly increase numbers of junctions and tubules. Using the ELISA, Holnthoner et al. (31) reported that an increasing amount of ADSCs in the co-culture resulted in the elevation of VEGF-A concentrations. It was mentioned that ADSCs were secreted a considerable amount of VEGF in the conditioned medium (22). Several studies indicated that the simultaneous co-stimulation of MPCs and endothelial tube formation were due to paracrine effects of VEGF as well as IGF-1, HGF, bFGF, and PDGF-BB (32, 33). On the contrary to our findings, Kook et al. (34) suggested that HUVECs co-cultured with ADSCs in the well plate did not observe any capillary formation. They reported that the proliferation, junctional proteins expression, and sprouts of HUVECs in the VEGF-loaded co-culture group were only slightly increased.

Unlike to results of research by Kook et al. (34), our finding study confirmed that ADSCs could induce endothelial tube formation in the VEGF-loaded co-culture group. It may be due to direct cellular interactions and, more importantly, the effects of paracrine secretion of ADSCs such as VEGF, angiopoietin-1, angiopoietin-2, and interleukin-6 that induce the proliferation and endothelial tube formation of HUVECs (35). The current research revealed that ADSCs conditioned medium (ADSCs-CM) had the potential to promote the vascular tube formation of HUVECs. *In vitro* and *in vivo* investigation revealed that the mesenchymal stem cell-conditioned media (MSC-CM) or ADSC-CM had a therapeutic effect with considerable results (36, 37).

ADSC-CM contained various growth factors such as VEGF, EGF, cytokines, proteins, and exosomes. Results of the present study were consistent with other research indicating that the ADSC-CM could improve the cell arrangement of endothelial cells. It is expected to enhance the proliferation and angiogenesis via paracrine effects of ADSC- CM (38, 39). Similar to finding of studies by Walter et al. (38) and Lee et al. (39), our study showed that the ADSC-CM could induce endothelial tube formation in VEGF-loaded group compared to HS and control groups. Due to the lack of these structures in other groups, it seems that an additional concentration of VEGF could induce endothelial tube formation in the VEGF-loaded group.

## Conclusion

It can be generally concluded that ADSCs/ HUVECs interaction and dual effects of VEGF can lead to the formation of differentiated myoblasts in proximity to endothelial network formations. Co-culture HUVECs and ADSCs can be a promising approach to achieve a favorable cellular design for tissue engineering of vascularized skeletal muscle. Furthermore, these *in vitro*-cellular models could be potentially used in vascular-muscle tissue engineering implanted into organ defects where muscle tissue and vascular regeneration were required.
